# Clinical and neurohormonal correlates of plasma TNF-α in a Zambian outpatient cohort

**DOI:** 10.1007/s44337-026-00738-x

**Published:** 2026-11-09

**Authors:** Arasidah Kaango, David Chisompola, Martin Chakulya, Emmanuel Luwaya, Dauyrinah Sidambi, Sydney Mulamfu, Benson M. Hamooya, Joreen P. Povia, Sepiso K. Masenga

**Affiliations:** 1 HAND Research Group, School of Medicine and Health Sciences, Mulungushi University, Livingstone, 10101 Zambia; 2 Department of Cardiovascular Science and Metabolic Diseases, Livingstone Center for Prevention and Translational Science, Livingstone, 10101 Zambia; 3 Department of Health Economics, Livingstone Center for Prevention and Translational Science, Livingstone, Zambia

**Keywords:** Tuberculosis, Inflammatory cytokines, HIV, Tumour necrosis factor-alpha, Interleukin-6, Interleukin-5, Immune activation

## Abstract

**Background:**

Chronic inflammation persists in populations with high HIV and tuberculosis (TB) burden, even during suppressive therapy. Tumor necrosis factor-alpha (TNF-α) is a key mediator, but its correlates beyond infectious status remain unclear in people living with HIV on stable antiretroviral therapy and who are virologically suppressed. We investigated clinical, metabolic, and neurohormonal correlates of TNF-α in a Zambian cohort.

**Methods:**

This was a cross-sectional study, which included 227 adults recruited from the outpatient medical clinic at Livingstone University Teaching Hospital in the Southern Province of Zambia. Simple and multivariable linear regression analyses were performed to determine independent associations with plasma TNF-α levels. Models were adjusted for HIV serostatus and history of TB. Standardized beta coefficients (β_std) were computed to compare the relative magnitude of associations across predictors measured on different scales.

**Results:**

Among 227 participants, 64.8% were living with HIV (95.3% on integrase strand transfer inhibitor-based ART) and 14.0% had a history of tuberculosis. In multivariable analysis, the strongest independent associations with TNF-α were observed within the renin-angiotensin-aldosterone system (RAAS): angiotensin II (β = 1.18 per pg/mL, *p* < 0.001; β_std = 0.51) and aldosterone (β = 0.884 per pg/mL, *p* < 0.001; β_std = 0.47). Living with HIV serostatus was also independently associated with higher TNF-α (β = 2581.1 pg/mL, *p* = 0.018; β_std = 0.19), while the history of TB was not. Significant metabolic factors associated with TNF-α levels included VLDL (β = 4397.4 per mmol/L, *p* = 0.038; β_std = 0.16) and triglycerides (β = 1971.6 per mmol/L, *p* = 0.034; β_std = 0.17). Inflammatory markers sST2 (β = 104.4 per ng/mL, *p* < 0.001; β_std = 0.31), IL-17 A (β = 1.137 per pg/mL, *p* = 0.003; β_std = 0.21), and D-dimer (β = 0.037 per pg/mL, *p* = 0.001; β_std = 0.22) were independently associated, as was plasma potassium (β = 107.3 per mmol/L, *p* < 0.001; β_std = 0.46). Renin, IL-6, IL-5, hs-CRP, and left ventricular mass were not associated with TNF-α.

**Conclusion:**

In this high-burden population, RAAS activation was an independent correlate of TNF-α, exceeding the effect of HIV serostatus. These findings support the hypothesis that RAAS inhibition may have anti-inflammatory effects; however, this remains to be tested in prospective and interventional studies.

**Supplementary Information:**

The online version contains supplementary material available at https://doi.org/10.1007/s44337-026-00738-x.

## Introduction

Tumor necrosis factor-alpha (TNF-α) is a key pro-inflammatory cytokine produced primarily by activated macrophages and T lymphocytes [1]. It plays a critical role in host defense against intracellular pathogens, including *Mycobacterium tuberculosis*, where it is essential for granuloma formation and containment of infection [2]. However, excessive or dysregulated TNF-α production contributes to systemic inflammation, endothelial dysfunction, and tissue injury.

This duality is particularly relevant in the context of HIV infection, where chronic immune activation sustains TNF-α signaling long after viral replication is controlled. Despite effective viral suppression with antiretroviral therapy (ART), people living with HIV (PLHIV) exhibit persistent immune activation characterized by elevated cytokines, coagulation markers, and endothelial dysfunction [3]. This chronic inflammatory state has been strongly linked to increased risk of cardiometabolic diseases, including atherosclerosis, hypertension, and insulin resistance [4]. This paradox is largely driven by persistent immune activation and chronic inflammation, which persist even in individuals achieving viral suppression on ART [5, 6].

Moreover, tuberculosis (TB) continues to pose a major threat. In 2020, the World Health Organization (WHO) reported TB as the leading cause of death from a single infectious agent globally, surpassing HIV/AIDS [7]. Although advances in chemoprophylaxis and TB treatment have been made, reducing TB incidence and mortality remains challenging, particularly in high-burden settings. TB-associated mortality is two to threefold higher in people living with HIV than in people living without HIV [8].

TNF-α is a central upstream cytokine that integrates innate and adaptive immune responses [1]. In PLHIV, TNF-α remains persistently elevated despite effective ART and has been strongly associated with endothelial dysfunction, immune activation, and cardiovascular risk [5]. Recent studies identify TNF-α as a robust biomarker reflecting residual inflammation and adverse clinical outcomes in treated HIV populations [9, 10].

Beyond its hemodynamic role, the renin–angiotensin–aldosterone system (RAAS) is increasingly recognized as a key regulator of inflammation [11]. Angiotensin II, acting primarily through the AT1 receptor, activates nuclear factor-κB (NF-κB) signaling, thereby promoting oxidative stress and stimulating the production of pro-inflammatory cytokines such as TNF-α in vascular endothelial cells, smooth muscle cells, and immune cells [12]. Similarly, aldosterone contributes to macrophage activation, vascular inflammation, and fibrosis processes that may exacerbate chronic immune activation and cardiovascular risk even in the setting of viral suppression [9, 11]. Importantly, RAAS dysregulation has been described in people living with HIV and linked to vascular dysfunction, metabolic abnormalities, and chronic immune activation [13, 14]. These observations provide a strong biological rationale for investigating RAAS components as correlates of TNF-α.

While persistent immune activation in treated HIV is well described, the relative contributions of neurohormonal, metabolic, and infectious factors remain incompletely understood, particularly in sub-Saharan African populations [15]. To our knowledge, few studies have simultaneously evaluated RAAS components alongside cytokine and metabolic markers to determine their independent contributions to systemic inflammation. We therefore aimed to investigate the independent clinical, metabolic, and neurohormonal correlates of circulating TNF-α levels, hypothesizing that RAAS activation would emerge as a significant determinant of TNF-α even after adjusting for HIV virologic status, TB co-infection, and other traditional factors.

## Materials and methods

### Study design and population

This study was designed as a cross-sectional analysis nested within an ongoing longitudinal cohort. The parent cohort comprised 366 adults recruited from the outpatient medical clinic at Livingstone University Teaching Hospital between 01 March 2025 and 01 September 2025, during routine clinical visits. For the present analysis, we included participants from the parent cohort who met predefined eligibility criteria, and consecutive sampling was applied, whereby all eligible individuals within the cohort during the study period were included to minimize selection bias. Eligibility for inclusion in the analytic sample required the availability of complete data for the primary outcome variable, serum tumor necrosis factor-alpha (TNF-α), as well as key covariates, including anthropometric and laboratory measurements. Participants with missing TNF-α data were excluded. Missing values were primarily attributable to inadequate serum sample volume, hemolysis, or laboratory processing limitations. Of the 366 participants in the parent cohort, 139 were excluded due to missing TNF-α measurements. The final analytic sample therefore consisted of 227 adults.

This study was reported in accordance with the Strengthening the Reporting of Observational Studies in Epidemiology (STROBE) guidelines for cross-sectional studies, and the completed STROBE checklist is provided as a supplementary file 1.

### Study setting

The study was conducted at Livingstone University Teaching Hospital, a tertiary referral facility for Southern and Western provinces of Zambia, which provides comprehensive care for people living with HIV and TB. Participants were recruited from the outpatient medical clinic, which manages approximately 3,800 patients on monthly basis. This clinic provides routine Health services including routine ART monitoring, counselling, and management of opportunistic infections, making it a suitable setting to investigate correlates of the pro-inflammatory cytokine TNF-α in a population highly burdened by HIV and TB.

### Eligibility criteria

Eligible participants were adults aged 18 years or older, provided written informed consent before enrolling on the study, living with HIV or participants without HIV and those with a history of TB/HIV co-infection. Among PLHIV, eligibility required receipt of stable antiretroviral therapy (ART) for at least 12 months and evidence of virological suppression, defined as a plasma HIV-1 RNA level < 50 copies/mL based on the most recent clinical measurement within the preceding 6 months. Participant living without HIV were required to have a documented negative HIV test. To minimize potential confounding effects on inflammatory markers, participants with conditions known to significantly influence systemic inflammation were excluded. These included diabetes mellitus, pregnancy, severe renal impairment (defined as estimated glomerular filtration rate [eGFR] < 30 mL/min/1.73 m^2^), severe hepatic disease (including cirrhosis or transaminase levels > 3 times the upper limit of normal), active opportunistic infections, autoimmune diseases, and other serious comorbid conditions.

### Sample size and sampling technique

A non-probability, consecutive sampling technique was employed, whereby all eligible individuals presenting to the study site during the recruitment period were invited to participate. This approach, while limiting generalizability, was chosen to efficiently enroll as many participants as possible from the target clinic population. A sample size calculation was performed for a multiple linear regression model. To detect a medium effect size (f^2^ = 0.15) with a significance level (α) of 0.05 and a statistical power of 90% for a model testing up to 12 predictors, a minimum sample size of *n* = 160 was required. To account for potential data issues and to ensure robust subgroup analysis, we targeted a larger sample. The final recruited cohort of *n* = 227 participants therefore provided ample statistical power to reliably identify the independent correlates of TNF-α reported in this analysis.

### Blood sampling conditions

Participants were initially recruited during routine medical outpatient clinic visits. Individuals who expressed interest and provided informed consent were scheduled for a separate study visit. They were instructed to fast for at least 8 h prior to the visit (from 8:00 PM the night before), during which only water was permitted. Participants returned to the clinic the following morning between 08:00 and 09:00 h, at which time blood samples were collected. Venipuncture was performed with participants in a seated position. Although fasting status and timing of sample collection were standardized, posture prior to sampling (e.g., duration of seated or supine rest) and dietary sodium intake were not formally controlled. Samples were processed according to standard laboratory protocols.

### Questionnaire development, pre-testing, and validation

A structured questionnaire was developed for this study to capture sociodemographic, clinical, medication, and behavioral data. Item generation was informed by existing literature on inflammation, HIV, TB, and cardiometabolic risk in sub-Saharan Africa. The initial draft contained 48 items across four domains: sociodemographic characteristics, clinical history, medication history, and behavioral factors. After it was developed, the questionnaire was entered into the Research Electronic Data Capture (REDCap) platform, with real-time validation rules to reduce data entry errors.

Content validity was assessed by a panel of five independent experts (two cardiologists, one infectious disease specialist, one epidemiologist/biostatistician, and one research scientist) who rated each item for relevance, clarity, and comprehensiveness. Based on the feedback obtained, two redundant items (self-reported diabetes and renal disease, already captured in clinical history and verified against medical records) were removed, and three items (employment status, smoking status, and marital status) were revised for clarity.

Pre-testing was conducted in a pilot study at the same outpatient clinic between 15 and 31 January 2025, prior to the main study. A convenience sample of 20 participants was recruited to reflect the target population: 10 people living with HIV (6 on INSTI-based ART, 4 on other regimens) and 10 people without HIV; 5 women and 5 men; age range 18–70 years. Participants were not included in the final analytic sample. The questionnaire was administered face-to-face by trained research assistants in the participant’s preferred language (Nyanja, Tonga, or English). Mean completion time was 22 min (range: 15–35 min).

### Sociodemographic and clinical data

Sociodemographic and clinical data were systematically collected and managed using Research Electronic Data Capture (REDCap) platform in Nyanja, Tonga, or English, as appropriate for the participant. The questionnaire captured the following domains: (1) sociodemographic characteristics such as age, sex, marital status (married/not married), and employment status (employed/not employed); (2) clinical history including HIV status, date of HIV diagnosis, antiretroviral therapy (ART) regimen and duration, history of tuberculosis (TB) diagnosis and treatment, history of hypertension, diabetes mellitus, and other chronic conditions; (3) medication history such as current antihypertensive medications, lipid-lowering agents, and other prescribed drugs; (4) behavioral factors such as smoking status (current/former/never). The questionnaire is provided as Supplementary file 2.

Trained research personnel gathered clinical data using a questionnaire, while anthropometric and laboratory measurements were collected using standardized study procedures.

### Anthropometric measurements

Height was measured to the nearest 0.1 cm using a calibrated stadiometer (SECA 213, SECA GmbH & Co. KG, Hamburg, Germany), with participants standing upright, heels together, and head positioned in the Frankfurt horizontal plane. Weight was measured to the nearest 0.1 kg using a calibrated digital scale (SECA 813, SECA GmbH & Co. KG, Hamburg, Germany), with participants wearing light clothing and no shoes. Body mass index (BMI) was calculated as weight in kilograms divided by height in meters squared (kg/m^2^). Waist circumference was measured to the nearest 0.1 cm at the midpoint between the lowest rib and the iliac crest using a non-elastic measuring tape (SECA 201, SECA GmbH & Co. KG, Hamburg, Germany). All measurements were performed by trained research personnel using standardized procedures, and each measurement was taken twice, with the average used for analysis.

### Biochemical measurements and outcome variable

The primary outcome variable for this study was plasma concentration of tumor necrosis factor-alpha (TNF-α). Venous blood samples were drawn from each participant via standard venipuncture into appropriate vacutainers including EDTA tubes where blood for hematological assays and plasma cytokine assays, heparin tubes for certain metabolic panels, and serum separator tubes for lipid and electrolyte analysis. For cytokines plasma was separated (for 15 min at 1000×*g* at 2–8℃ within 30 min of collection) and stored at − 80 °C until analysis.

IL-17 A, IL-5, IL-6, TNF-α, and IFN-γ were quantified using an enzyme-linked immunosorbent assay (ELISA) commercially available kits Elabscience (Elabscience Biotechnology Inc., Houston, USA), following manufacturer protocols. Calibration curves were generated for each assay plate using serial dilutions of standard controls. The detection ranges for cytokines were as follows: TNF-α (Catalog No: E-EL-H0109, with the detection range of 7.81–500 pg/mL), IL-5 (Catalog No: E-EL-H0191, with the detection range of 15.63–1000 pg/mL), IL-6 (Catalog No: E-EL-H6156, with the detection range of 1.56–100 pg/mL), IL-17 A (Catalog No: E-EL-H5812, with the detection range of 7.81–500 pg/mL) and IFN-γ (Catalog No: E-EL-H0108, with the detection range of 15.63–1000 pg/mL). Samples with concentrations exceeding the upper limit of the respective calibration range were appropriately diluted, re-assayed, and concentrations were calculated by multiplying the concentration obtained from the standard curve by the corresponding dilution factor. The intra-assay coefficients of variation (CVs) were < 8%, and inter-assay CVs were < 10%, consistent with acceptable assay performance thresholds reported in biomarker studies (typically < 10%).

Lipid profile [triglycerides, LDL-cholesterol, HDL-cholesterol, Very low-density lipoprotein (VLDL)], and plasma potassium were measured using standard automated clinical chemistry analyzers (Cobas c111, Roche Diagnostics, Mannheim, Germany) following manufacturer protocols.

Laboratory analyses were conducted in a single laboratory, and all samples were analyzed in batches to reduce inter-assay variability.

### Left ventricular mass measurement

Left ventricular mass was calculated from 2D echocardiography using the validated Devereux cube formula:

  

$${\mathrm{LVM}}=0.8 \times 1.04 \times \left[ {\left( {{\mathrm{LVIDd}}\,+\,{\mathrm{IVSd}}\,+\,{\mathrm{LVPWd}}} \right) 3-{\mathrm{LVIDd}}3} \right]+0.6\;{\mathrm{g}},$$

where IVSd is the interventricular septal thickness at end-diastole, LVIDd is the left ventricular internal diameter at end-diastole, and PWTd is the posterior wall thickness at end-diastole. Echocardiographic assessments were performed using a DW-P30 Color Doppler Ultrasound System (Dawei Medical, Economic Technology and Development Zone, Xuzhou, Jiangsu, China) equipped with a 2.0–4.0 MHz phased-array sector transducer. All measurements were performed by a single experienced cardiologist blinded to clinical data, according to the American Society of Echocardiography guidelines [16].

### Statistical analysis

Data were exported from the REDCap platform into a structured format and underwent initial cleaning in Microsoft Excel (Microsoft Corporation, Redmond, WA, USA; version 2021) to ensure data integrity. All statistical analyses were performed using StatCrunch (Pearson Education, Inc., Boston, MA, USA; available at https://www.statcrunch.com/), a web-based statistical software application. The distribution of continuous variables was assessed using the Shapiro–Wilk test for normality. Several biomarkers, including TNF-α, IL-6, IL-5, IL-17 A, sST2, hs-CRP, D-dimer, aldosterone, renin, and angiotensin II, exhibited right-skewed distributions (Supplementary file 3). Log10 and natural log transformations were considered. However, because ordinary least squares regression assumes normality of residuals rather than of predictors or the outcome, and because residual diagnostics for the primary models were acceptable, untransformed variables were retained in the primary analysis to preserve clinical interpretability of beta coefficients (expressed per pg/mL or per mmol/L). To facilitate comparison of the relative magnitude of associations, standardized beta coefficients (β_std) were computed and are reported alongside unstandardized coefficients [17] (Supplementary file 4). Descriptive statistics were presented as medians with interquartile ranges (IQR) for continuous variables based on the results of the normality assessment, and frequencies with percentages for categorical variables. The relationship between TNF-α and associated biomarkers was first assessed using simple linear regression, with results visualized in a correlogram and forest plots. Variables showing a significance level of *p* < 0.05 in the bivariate analysis were included in the multivariable linear regression models. Second, a priori confounders were retained regardless of bivariate significance based on biological plausibility and prior literature, including age, sex, body mass index, smoking status, HIV serostatus, and history of tuberculosis [18]. This approach is consistent with recommended practices for confounder selection in observational studies, which prioritize a priori knowledge over purely data-driven selection to reduce bias. In a sensitivity analysis using log-transformed TNF-α and log-transformed skewed predictors, the direction, statistical significance, and relative magnitude of all primary associations were unchanged (Supplementary file 5).

A p-value of < 0.05 was considered statistically significant in the multivariable models.

Multicollinearity among RAAS variables was assessed using variance inflation factors (VIFs), with values > 10 indicating potential collinearity. In addition, pairwise correlations between RAAS biomarkers were examined. No evidence of problematic multicollinearity was observed.

The distribution of continuous variables was assessed prior to analysis using graphical methods (histograms and Q–Q plots) and formal normality tests, including the Shapiro–Wilk test. No transformations were applied, as variables included in the final models satisfied the assumptions of the statistical methods used.

In sensitivity analyses restricted to people living with HIV, multivariable models were adjusted for the same set of covariates as in the primary analysis, including age, sex, BMI, smoking status, blood pressure, kidney function, and HIV-related factors, to ensure consistency and allow comparability of estimates.

Analyses were conducted using complete-case data for each model; therefore, the number of observations may vary depending on the availability of data for included variables. Complete-case analysis was chosen a priori because missing TNF-α values were predominantly attributable to technical laboratory factors (insufficient serum volume, hemolysis, or processing limitations) rather than participant characteristics, which is plausibly consistent with a missing-completely-at-random mechanism; however, we did not formally compare included and excluded participants, and we cannot definitively verify this assumption. The use of complete-case analysis, and the absence of a formal comparison between included and excluded participants, are acknowledged as limitations.

### Variables included in the analysis

Continuous variables: Age (years), body mass index (kg/m^2^), waist circumference (cm), plasma TNF-α (pg/mL), IL-6 (pg/mL), IL-17 A (pg/mL), IL-5 (pg/mL), IFN-γ (pg/mL), hs-CRP (pg/mL), D-dimer (pg/mL), sST2 (ng/mL), plasma potassium (mmol/L), spot microalbumin (mg/L), renin (pg/mL), aldosterone (pg/mL), angiotensin II (pg/mL), VLDL (mmol/L), triglycerides (mmol/L), LDL-cholesterol (mmol/L), HDL-cholesterol (mmol/L), LDL/HDL ratio, total cholesterol/HDL ratio, mean corpuscular volume (fL), and left ventricular mass (g).

Categorical variables: Sex (male/female), marital status (married/not married), employment status (employed/not employed), HIV serostatus (living with HIV/living without HIV), ART regimen (NNRTI+NRTI/INSTI/PIs), history of tuberculosis (yes/no), hypertension status (hypertensive/non-hypertensive), hypertensive heart disease (yes/no), and smoking status (current smoker/non-smoker).

Continuous variables were analyzed per unit increase as indicated, and categorical variables are presented relative to the reference group (male sex; married; employed; living without HIV; no history of TB; non-hypertensive; non-smoker).

## Results

### Basic characteristics of the study population

A total of 227 study participants were recruited of which the median age was 50 years (IQR: 40–59), and the cohort was predominantly female (67.8%), with more than half of participants unmarried (55.8%) and unemployed (66.4%). Most participants were living with HIV (64.8%), while 35.2% were without HIV; among those receiving antiretroviral therapy, 95.3% were on integrase strand transfer inhibitor–based regimens (TLD or TafED). A prior history of tuberculosis was reported by 14.0% of participants with available data. Hypertension was present in 29.1% of the cohort, and 10.9% had hypertensive heart disease, while current smoking was uncommon (4.6%). The median body mass index was 25.4 kg/m^2^, indicating a tendency toward overweight, with median triglyceride levels of 0.8 mmol/L and an HDL-cholesterol ratio of 3.71. Inflammatory biomarkers were markedly elevated, with high median concentrations of hs-CRP (73,649.3 pg/mL), IL-6 (86.1 pg/mL), and other cytokines including IL-17 A and IL-5, showing wide interindividual variability. Indicators of cardiac and renal stress were also evident, with a median left ventricular mass of 144.8 g and a median spot micro albumin of 21.5 mg/dL. Components of the renin–angiotensin–aldosterone system were substantially activated, as reflected by high median renin (2,390.3 pg/mL) and aldosterone (165.4 pg/mL) levels, accompanied by considerable variability across participants (Table 1).

**Table 1:** Baseline characteristics of the study population (*n* = 227)

Variable	Frequency (*n*)	Percent (%)
Age (years), IQR	50 (40, 59)	
Sex		
Male	73	32.2
Female	154	67.8
Marital status		
Married	100	44.2
Not married	126	55.8
Employment status		
Employed	76	33.6
Not employed	150	66.4
HIV status		
Living with HIV	147	64.8
Living without HIV	80	35.2
ART regimen		
NNRTI+NRTI	4	3.1
INSTI (TLD or TafED)	122	95.3
PIs	2	1.6
History of TB		
Yes	26	14.0
No	160	86.0
Hypertension status		
Hypertensive	66	29.1
Non-hypertensive	161	70.9
Hypertensive heart disease		
Yes	23	10.9
No	188	89.1
Smoking		
Yes	10	4.6
No	208	95.4
BMI (kg/m^2^), IQR	25.4 (21.0, 29.4)	
IL-6 (pg/mL), IQR	86.1 (0.80, 108.8)	
IL-17 A (pg/mL), IQR	22.9 (18.9, 491.3)	
hs-CRP, (pg/mL), IQR	73649.3 (9211.1, 153333.9)	
MCV (fL), IQR	93.6 (88.1, 98.6)	
IL-5 (pg/mL) IQR	456.2 (13.3, 1370.8)	
Soluble sT2, ng/mL, IQR	2.8 (0.26, 29.3)	
Plasma potassium (mmol/L), IQR	4.0 (3.8, 4.3)	
Spot microalbumin, (mg/L), IQR	21.5 (9.6, 42.8)	
Aldosterone, (pg/mL), IQR	165.4 (85.5, 6192.1)	
Renin, (pg/mL), IQR	2390.3 (22.0, 3292.3)	
Angiotensin, (pg/mL), IQR	1691.8 (42.3, 4884.9)	
VLDL(mmol/L), IQR	0.36 (0.25, 0.54)	
Cholesterol HDL ratio, IQR	3.71 (3.0, 4.6)	
LDL HDL ratio, IQR	2.5 (1.87, 3.47)	
Triglycerides (mmol/L), IQR	0.8 (0.55, 1.18)	
LVMass (g), IQR	144.8 (120.8, 183.4)	

*IQR* interquartile range, *ART* antiretroviral therapy, *NNRTI* non-nucleoside reverse transcriptase inhibitor, *NRTI* nucleoside reverse transcriptase inhibitor, *INSTI* integrase-strand transfer inhibitor, *TLD* Tenofovir/Lamivudine/Dolutegravir, *TafED* Tenofovir alafenamide/Emtricitabine/Dolutegravir, *TB* Tuberculosis, *CRP* C-reactive protein, *HDL* high-density lipoprotein, *LDL* low-density lipoprotein

**Fig. 1:**
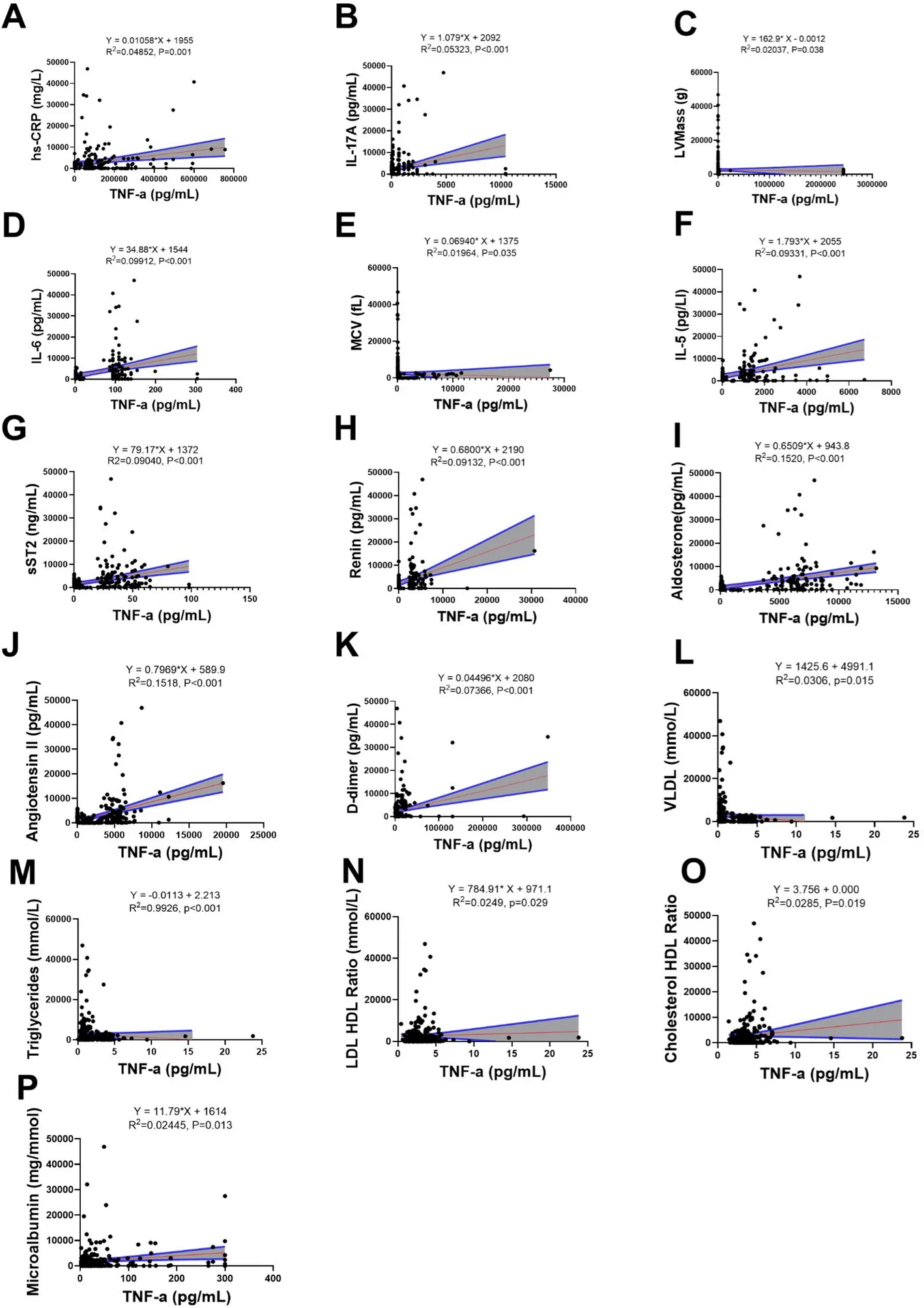
Unadjusted associations of clinical, metabolic, inflammatory and neurohormonal factors with plasma TNF-α concentrations

Figure 1 shows significant positive associations between TNF-α levels and multiple inflammatory cytokines, including IL-17 A, IL-6, TNF-α, IL-5, and sST2, indicating coordinated immune activation. IFN-γ was also positively correlated with hs-CRP, IgE, and components of the renin–angiotensin–aldosterone system (renin, aldosterone, and angiotensin II). Modest but significant associations were observed with fasting glucose, systolic blood pressure variability, vascular parameters, and spot microalbumin (Fig. 1).

### Simple linear regression of factors associated with TNF-α

In unadjusted linear regression analyses, several clinical, metabolic, and inflammatory variables were significantly associated with TNF-α concentrations (Table 2). When standardized beta coefficients were examined to compare the relative magnitude of associations, plasma potassium (β_std = 0.46), angiotensin II (β_std = 0.44), and aldosterone (β_std = 0.42) exhibited the largest standardized effects. Living with HIV status was positively associated with TNF-α levels (β = 2573.3, 95% CI 803.9 to 4342.7; *p* = 0.005; β_std = 0.19). Lipid-related markers, including VLDL (β = 4991.1; *p* = 0.015; β_std = 0.19), triglycerides (β = 2238.6; *p* = 0.013; β_std = 0.19), LDL/HDL ratio (β = 971.1; *p* = 0.029; β_std = 0.16), and total cholesterol/HDL ratio (β = 974.0; *p* = 0.019; β_std = 0.18), were also significantly associated with increased TNF-α (Table 2). Markers of RAAS activation showed consistent and strong associations, including angiotensin II (β = 1.0; *p* < 0.001; β_std = 0.44), aldosterone (β = 0.8; *p* < 0.001; β_std = 0.42), and renin (β = 0.7; *p* < 0.001; β_std = 0.34). Similarly, inflammatory biomarkers such as IL-6 (β = 43.2; *p* < 0.001; β_std = 0.38), IL-5 (β = 2.0; *p* < 0.001; β_std = 0.36), sST2 (β = 108.8; *p* < 0.001; β_std = 0.33), D-dimer (β = 0.04; *p* < 0.001; β_std = 0.24), IL-17 A (β = 1.3; *p* < 0.001; β_std = 0.24), and hs-CRP (β = 0.01; *p* = 0.003; β_std = 0.22) were all positively associated with TNF-α). In contrast, mean corpuscular volume (MCV) and left ventricular mass (LVMass) demonstrated inverse associations with TNF-α. Plasma potassium levels showed a strong positive association (β = 108.9; *p* < 0.001; β_std = 0.46), further supporting a potential link between electrolyte balance and inflammatory activity. These findings informed the selection of variables included in subsequent multivariable analyses (Table 2).

**Table 2:** Unadjusted linear regression analysis of factors associated with TNF-α concentrations

Parameter	Beta (β)	β_std	95% L. Limit	95% U. Limit	*p*-value
Age, years	53.6	0.11	− 8.2	115.5	0.089
Sex (Female)	629.9	0.05	− 1052.4	5360.8	0.187
Body mass index (Kg/m^2^)	− 128.1	− 0.11	− 281.6	25.3	0.101
Smoking status (Smoker)	− 1647.2	− 0.05	− 5896.7	2602.3	0.446
History of TB (Yes)	426.9	0.02	− 2315.7	3169.5	0.759
HIV status (Living with HIV)	2573.3	0.19	803.9	4342.7	**0.005**
VLDL (mmol/L)	4991.1	0.19	994.3	8987.9	**0.015**
Triglycerides (mmol/L)	2238.6	0.19	486.7	3990.6	**0.013**
LDL HDL Ratio	971.1	0.16	102.2	1840.1	**0.029**
Cholesterol HDL ratio	974.0	0.18	164.0	1784.1	**0.019**
Angiotensin II (pg/mL)	1.0	0.44	0.7	1.3	**< 0.001**
Aldosterone (pg/mL)	0.8	0.42	0.6	1.1	**< 0.001**
Renin (pg/mL)	0.7	0.34	0.3	1.0	**< 0.001**
hs-CRP (pg/mL)	0.01	0.22	0.004	0.021	**0.003**
D-dimer (pg/mL)	0.04	0.24	0.02	0.06	**< 0.001**
IL-17 A (pg/mL)	1.3	0.24	0.6	2.0	**< 0.001**
IL-6 (pg/mL)	43.2	0.38	23.9	62.7	**< 0.001**
IL-5 (pg/mL)	2.0	0.36	1.0	2.9	**< 0.001**
sST2 (pg/mL)	108.8	0.33	68.5	149.0	**< 0.001**
MCV (fL)	− 101.0	− 0.19	− 180.2	− 21.7	**0.013**
Plasma Potassium (mmol/L)	108.9	0.46	78.8	139.0	**< 0.001**
Spot microalbumin (mg/L)	13.7	0.13	0.5	26.9	**0.042**
LVMass (g)	− 16.9	− 0.15	− 32.9	− 0.9	**0.038**

β = unstandardized coefficient (change in TNF-α in pg/mL per unit increase in predictor). β_std = standardized coefficient (change in SD of TNF-α per 1 SD increase in predictor). SD of TNF-α = 6576.3 pg/mL. Categorical variables were not standardized

*TNF-α* tumor necrosis factor-alpha, *VLDL* very low-density lipoprotein, *LDL* low-density lipoprotein, *HDL* high-density lipoprotein, *hs-CRP* high-sensitivity C-reactive protein, *IL* interleukin, *sST2* soluble suppression of tumorigenicity-2, *MCV* mean corpuscular volume, *LVMass* left ventricular mass

β coefficients represent the change in TNF-α concentration per unit increase in each predictor. Confidence intervals (CI) are reported at the 95% level. p-values were derived from unadjusted linear regression models. Continuous variables were analyzed per unit increase as indicated, and categorical variables are presented relative to the reference group. Statistical significance was defined as *p* < 0.05 and p values <0.05 are in bold

### Factors associated with TNF-α

Multiple linear regression analysis identified specific metabolic, inflammatory, and neurohormonal factors as independent correlates of plasma TNF-α levels by adjusting with HIV and History of TB (Table 3). The strongest positive associations were observed within RAAS. A 1 pg/mL increase in angiotensin II was associated with a 1.18 pg/mL increase in TNF-α (Beta (β) = 1.18, 95% CI 0.791 to 1.577, *p* < 0.001; β_std = 0.51). Similarly, a 1 pg/mL increase in aldosterone was associated with a 0.884 pg/mL increase in TNF-α (β = 0.884, 95% CI 0.588 to 1.180, *p* < 0.001 β_std = 0.47). Plasma potassium also showed a strong independent association, with each 1 mmol/L increase associated with a 107.3 pg/mL increase in TNF-α (β = 107.3, 95% CI 78.35 to 136.2, *p* < 0.001; β_std = 0.46). Living with HIV serostatus was independently associated with elevated TNF-α (β = 2581.1 pg/mL, 95% CI 439.4 to 4722.7, *p* = 0.018; β_std = 0.19). Among lipid parameters, higher VLDL (β = 4397.4, 95% CI 241.1 to 8553.7, *p* = 0.038; β_std = 0.16) and triglyceride levels (β = 1971.6, 95% CI 152.7 to 3790.4, *p* = 0.034 ; β_std = 0.17) were significantly associated with elevated TNF-α. Several inflammatory and cardiac stress biomarkers were also significant predictors. Each 1 ng/mL increase in soluble ST2 (sST2) was associated with a 104.4 pg/mL increase in TNF-α (β = 104.4, 95% CI 48.16 to 160.7, *p* < 0.001; β_std = 0.31). IL-17 A (β = 1.137, 95% CI 0.397 to 1.876, *p* = 0.003; β_std = 0.21) and D-dimer (β = 0.037, 95% CI 0.014 to 0.059, *p* = 0.001 ; β_std = 0.22) showed positive associations.

Notably, History of TB was not a significant predictor of TNF-α levels in the adjusted model (β = − 443.96, 95% CI − 2958.133 to 2070.2, *p* = 0.722; β_std = − 0.02). Other variables, including age, sex, body mass index, smoking status, renin, IL-6, IL-5, hs-CRP, cholesterol ratios, mean corpuscular volume, spot micro albumin, and left ventricular mass, did not show statistically significant independent associations at the *p* < 0.05 threshold.

**Table 3:** Multivariable linear regression of factors associated with TNF-α concentrations

Parameter	Beta (β)	β_std	95% L. Limit	95% U. Limit	*p*-value
Age, years	19.3	0.04	− 58.7	97.4	0.626
Sex (Female)	329.8	0.02	− 1677.5	2337.2	0.746
Body mass index (Kg/m^2^)	− 158.2	− 0.14	− 332.9	16.4	0.075
Smoking status (Smoker)	− 2182.5	− 0.07	− 6907.2	2542.0	0.363
History of TB (Yes)	− 443.962	− 0.02	− 2958.133	2070.2	0.722
HIV status (Living with HIV)	2581.1	0.19	439.4	4722.7	**0.018**
VLDL (mmol/L)	4397.4	0.16	241.1	8553.7	**0.038**
Triglycerides (mmol/L)	1971.6	0.17	152.7	3790.4	**0.034**
LDL HDL Ratio	779.9	0.13	− 153.2	1713.0	0.101
Cholesterol HDL ratio	803.1	0.15	− 71.377361	1677.6	0.072
Angiotensin II (pg/mL)	1.18	0.51	0.791	1.577	**< 0.001**
Aldosterone (pg/mL)	0.884	0.47	0.588	1.180	**< 0.001**
Renin (pg/mL)	0.276	0.14	− 0.166	0.717	0.219
hs-CRP (pg/mL)	0.0085	0.19	0.0008	0.0178	0.073
D-dimer (pg/mL)	0.037	0.22	0.014	0.059	**0.001**
IL-17 A (pg/mL)	1.137	0.21	0.397	1.876	**0.003**
IL-6 (pg/mL)	12.98	0.11	− 28.64	54.61	0.538
IL-5 (pg/mL)	0.671	0.12	− 0.716	2.058	0.340
sST2 (pg/mL)	104.4	0.31	48.16	160.7	**< 0.001**
MCV (fL)	− 77.62	− 0.14	− 179.2	23.98	0.133
Plasma Potassium (mmol/L)	107.3	0.46	78.35	136.2	**< 0.001**
Spot microalbumin (mg/L)	12.24	0.12	− 4.943	29.42	0.161
LVMass (g)	− 16.55	− 0.14	− 34.93	1.832	0.077

β = unstandardized coefficient (change in TNF-α in pg/mL per unit increase in predictor). β_std = standardized coefficient (change in SD of TNF-α per 1 SD increase in predictor). SD of TNF-α = 6576.3 pg/mL. Categorical variables were not standardized. Model adjusted for age, sex, BMI, smoking status, HIV serostatus, and history of TB. *P* < 0.05 indicates significance and the p values are in bold

*HIV* Human Immunodeficiency Virus, *VLDL* Very Low-Density Lipoprotein, *LDL/HDL* ratio of Low- to High-Density Lipoprotein, *hs-CRP* high-sensitivity C-Reactive Protein, *IL* Interleukin, *sST2* Soluble Suppression of Tumorigenicity 2, *MCV* Mean Corpuscular Volume, *LVMass* Left Ventricular Mass

### Sensitivity analysis between RAAS biomarkers and TNF-α among people living with HIV

In sensitivity analyses restricted to people living with HIV and adjusted for an expanded set of clinical, metabolic, and inflammatory variables, the associations between RAAS biomarkers and TNF-α remained robust. Higher angiotensin II and aldosterone levels were independently associated with increased TNF-α (β = 1.20, 95% CI 0.73–1.67, *p* < 0.001; β_std = 0.40 and β = 0.88, 95% CI 0.53–1.23, *p* < 0.001; β_std = 0.39. These findings were consistent with the primary analysis, supporting the stability of the observed relationships (Table 4).

**Table 4:** Sensitivity analysis: multivariable association between RAAS biomarkers and TNF-α among people living with HIV

Parameter	Beta (β)	β_std	95% L. Limit	95% U. Limit	*p*-value
Age, years	34.1	0.05	− 87.8	156.2	0.580
Sex (Female)	843.9	0.00	− 1996.6	3684.5	0.558
Body mass index (Kg/m^2^)	− 218.2	− 0.15	− 465.7	29.1	0.083
Smoking status (Smoker)	− 2182.5	− 0.07	− 7795.5	3430.3	0.443
History of TB (Yes)	− 444.2	− 0.02	− 3760.8	2872.4	0.791
VLDL (mmol/L)	5620.7	0.18	13.3	11228.1	**0.049**
Triglycerides (mmol/L)	2467.5	0.19	26.7	4908.3	**0.048**
LDL HDL Ratio	1089.3	0.15	− 234.6	2413.4	0.106
Cholesterol HDL ratio	1130.5	0.17	− 119.2	2380.2	0.076
Angiotensin II (pg/mL)	1.20	0.40	0.73	1.67	**< 0.001**
Aldosterone (pg/mL)	0.88	0.39	0.53	1.23	**< 0.001**
Renin (pg/mL)	0.27	0.12	− 0.28	0.83	0.331
hs-CRP (pg/mL)	0.01	0.18	− 0.001	0.018	0.077
D-dimer (pg/mL)	0.03	0.18	0.01	0.06	**0.007**
IL-17 A (pg/mL)	1.13	0.21	0.25	2.01	**0.011**
IL-6 (pg/mL)	14.9	0.07	− 40.7	70.5	0.595
IL-5 (pg/mL)	0.67	0.10	− 1.09	2.43	0.451
sST2 (pg/mL)	104.9	0.27	38.1	171.7	**0.002**
MCV (fL)	− 103.3	− 0.15	− 242.4	35.7	0.143
Plasma Potassium (mmol/L)	107.2	0.48	72.5	142.1	**< 0.001**
Spot microalbumin (mg/L)	12.7	0.11	− 9.0	34.5	0.247
LVMass (g)	− 27.4	− 0.17	− 55.5	0.62	0.055

This model includes an expanded set of covariates to assess robustness of associations. Variables were selected based on biological relevance; however, given the number of covariates relative to sample size, results should be interpreted cautiously. p valeus <0.05 are in bold

### Forest plot of factors associated with TNF-α

**Fig. 2:**
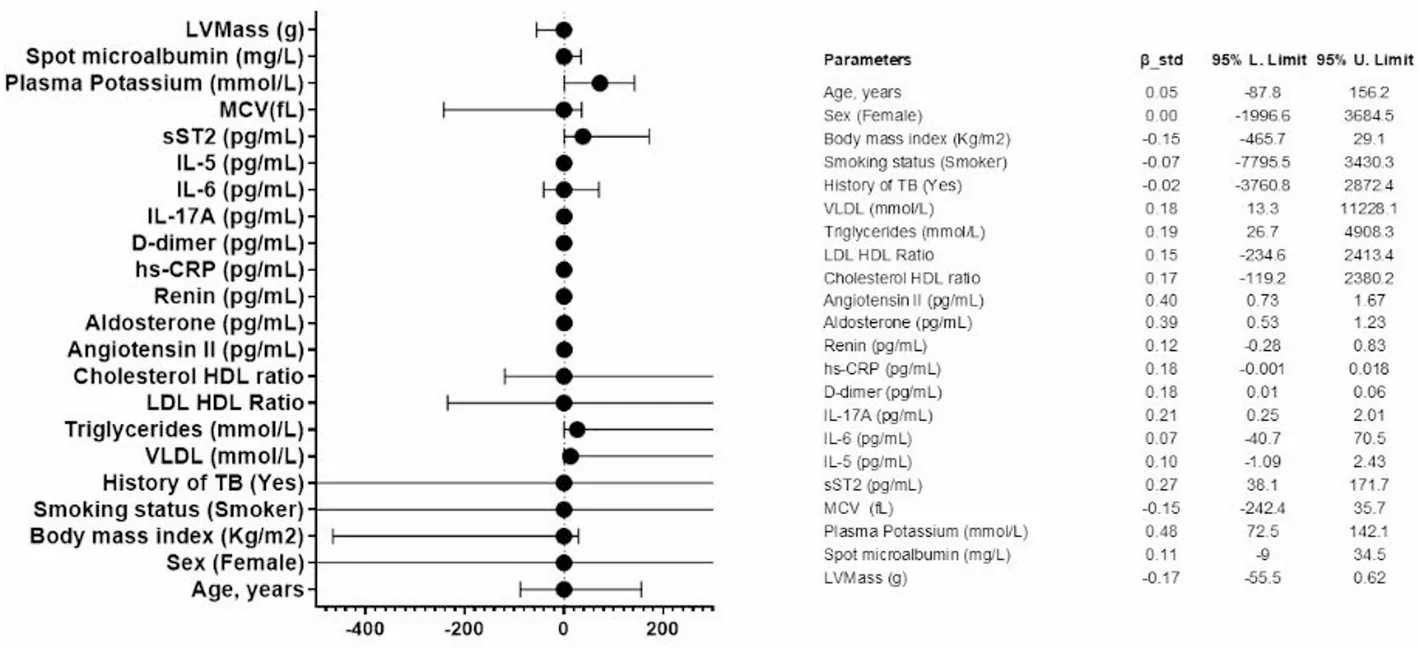
Forest plot of factors associated with TNF-α

Figure 2 Forest plot showing the associations between clinical, metabolic, inflammatory, and RAAS variables and the study outcome derived from multivariable regression analysis. Points represent standardized beta (β) coefficients, and horizontal lines indicate 95% confidence intervals (CI), plotted on a logarithmic scale. Variables include HIV status, history of tuberculosis, lipid parameters (VLDL, triglycerides, LDL/HDL ratio, cholesterol/HDL ratio), inflammatory markers (hs-CRP, D-dimer, IL-17 A, IL-6, IL-5), cardiac and renal indices (left ventricular mass [LVMass], spot microalbumin), hematological and electrolyte measures (MCV, plasma potassium), and RAAS components (renin, aldosterone, angiotensin II), as well as sST2. Statistical significance: HIV status (*p* = 0.018), VLDL (*p* = 0.038), Triglycerides (*p* = 0.034), Angiotensin II (*p* < 0.001), Aldosterone (*p* < 0.001), D-dimer (*p* = 0.001), IL-17 A (*p* = 0.003), sST2 (*p* < 0.001), and plasma potassium (*p* < 0.001) were significant; history of tuberculosis, renin, IL-6, IL-5, hs-CRP, and others were not (all *p* > 0.05).

## Discussion

This study demonstrates that, in a high HIV/TB burden population with predominantly virally suppressed individuals, TNF-α levels are independently associated with both HIV serostatus and neurohormonal/cardiometabolic pathways, with angiotensin II and aldosterone showing the strongest quantitative effects respectively. These findings contribute to the growing body of evidence highlighting persistent immune activation in populations affected by HIV, TB, and their co-infection, even in the era of expanded ART [19].

The observed associations between RAAS biomarkers and TNF-α are biologically plausible and supported by mechanistic evidence. Angiotensin II, through activation of the angiotensin II type 1 receptor (AT1R), stimulates intracellular signaling pathways including nuclear factor-kappa B (NF-κB), a key transcription factor regulating pro-inflammatory cytokines such as TNF-α, IL-6, and IL-1β [20–22]. In addition, angiotensin II promotes oxidative stress through the generation of reactive oxygen species, which further amplifies NF-κB activation and inflammatory signaling [23, 24]. Aldosterone, via mineralocorticoid receptor activation, also contributes to inflammation by promoting macrophage activation, vascular injury, and fibrosis [25]. These pathways provide a mechanistic basis linking RAAS activation to systemic inflammation, and may be particularly relevant in treated HIV populations characterized by persistent immune activation.

While angiotensin II and aldosterone were associated with higher TNF-α levels, these findings should be interpreted as associations rather than evidence of causality. Interactions between RAAS activation and inflammatory pathways are complex and likely bidirectional, with angiotensin II promoting cytokine production, including TNF-α, while inflammatory cytokines may also influence RAAS activity. These results therefore reflect relationships within a broader inflammatory network rather than a unidirectional regulatory pathway.

In our simple linear regression analysis, TNF-α emerged as part of a broader, tightly coordinated pro-inflammatory cytokine rather than acting as an isolated immune mediator. Moreover, In multivariable models, the pattern of associations with TNF-α became highly selective identifying neurohormonal and cardiometabolic factors as the most important independent correlates [26, 27]. Similar patterns of cytokine co-activation have been reported in TB and HIV/TB co-infection, where TNF-α plays a central role in granuloma maintenance but may also contribute to pathological inflammation when excessively expressed [28].

The observed associations between TNF-α and HIV may have important implications for cardiometabolic risk among people living with HIV. Despite effective antiretroviral therapy, people living with HIV exhibit persistent immune activation and chronic inflammation, which are important contributors to cardiovascular disease [29]. Emerging evidence suggests that dysregulation of the renin–angiotensin–aldosterone system contributes to this process by promoting endothelial dysfunction, oxidative stress, and pro-inflammatory cytokine production [30]. Elevated TNF-α levels have been linked to increased cardiovascular risk in people living with HIV, including accelerated atherosclerosis. Our findings raise the possibility that RAAS activation represents a mechanistic link between immune activation and cardiometabolic complications in treated HIV populations. These pathways may also have therapeutic relevance, given evidence that pharmacological inhibition of the RAAS can attenuate inflammation and improve cardiovascular outcomes.

The metabolic correlates VLDL and triglycerides, align with growing evidence linking dyslipidemia to inflammation [31, 32]. VLDL particles and triglycerides play crucial, yet distinct, roles in promoting vascular inflammation and insulin resistance. Emerging research supports that VLDL can directly stimulate inflammatory responses in macrophages, while triglyceride levels serve as a primary biomarker for systemic metabolic dysfunction [33]. These findings suggest that, in this high HIV and TB burden setting, TNF-α may reflect downstream integration of neurohormonal, metabolic and immune–thrombotic pathways rather than direct effects of infectious status alone. While HIV serostatus retains significance, the magnitude of RAAS associations suggests neurohormonal pathways may play an important role within the broader inflammatory network in this context.

The inflammatory markers that retained independent associations, sST2, IL-17 A, and D-dimer, point to distinct but interconnected pathways. sST2, a decoy receptor for IL-33, reflects cardiac stress and fibrosis and has emerged as a powerful predictor of cardiovascular outcomes [34]. Beyond its role as a biomarker, the IL-33/ST2 pathway modulates immune cell activation and may amplify TNF-α–mediated inflammatory signaling under conditions of tissue stress. IL-17 A, produced by Th17 cells, is implicated in autoimmune and chronic inflammatory conditions and may link adaptive immunity to tissue inflammation [35]. IL-17 A promotes neutrophil recruitment, endothelial activation, and synergistic induction of pro-inflammatory cytokines including TNF-α, suggesting a feed-forward amplification loop between innate and adaptive immune pathways. Meanwhile, D-dimer, a fibrin degradation product, indicates coagulation activation and has been associated with mortality in both HIV and non-HIV populations [36].

The observed association between plasma potassium levels and TNF-α may reflect underlying inflammatory regulatory pathways. One potential mechanism involves the NLRP3 inflammasome, which is known to be activated by potassium efflux and plays a central role in innate immune signaling [37]. Activation of this pathway leads to the production of pro-inflammatory cytokines such as IL-1β and IL-18, and may interact with broader inflammatory networks that include TNF-α. However, it is important to emphasize that inflammasome components and downstream cytokines were not measured in this study. Therefore, this interpretation should be considered hypothesis-generating. Further studies incorporating direct assessment of inflammasome activation are required to clarify the mechanistic relationship between potassium homeostasis and inflammatory signaling in this population.

Although IL-6 is a key inflammatory cytokine with well-established interactions with TNF-α, it did not remain independently associated with the outcome in multivariable analysis. This finding may reflect shared upstream regulatory pathways and collinearity among inflammatory mediators, whereby the independent contribution of IL-6 is attenuated after adjustment for related variables. In addition, IL-6 exhibits context-dependent biological effects, functioning in both pro- and anti-inflammatory roles, and is characterized by substantial inter-individual variability. These properties may reduce its independent predictive value in multivariable models. It is also possible that RAAS-related mechanisms, which demonstrated strong associations in this study, interact more directly with TNF-α–mediated pathways, thereby contributing to the observed pattern. These findings highlight the complexity of inflammatory signaling networks and suggest that TNF-α may serve as a more robust marker of inflammation in this cohort.

The findings of the study have important clinical implications. The strong association between TNF-α and RAAS components suggests that neurohormonal blockade may have anti-inflammatory effects beyond blood pressure control [38]. Furthermore, the links between TNF-α, dyslipidemia, coagulation markers, and cardiac stress biomarkers highlight TNF-α as a potential integrative marker of cardiometabolic inflammation. This may be particularly relevant in populations with overlapping infectious, metabolic, and cardiovascular risk profiles [39].

This study has several notable strengths. It was conducted in a high HIV/TB burden setting, addressing an important gap in the literature. The integration of neurohormonal and inflammatory biomarkers provides novel insight into potential mechanisms linking RAAS activation and immune activation. In addition, the use of multivariable statistical models allowed for the identification of independent associations. Finally, the study draws on data from a well-characterized African cohort, contributing valuable regional evidence to an area that remains underrepresented in global research.

This study has several limitations that should be considered. First, the cross-sectional design precludes causal inference, and the temporal relationship between RAAS activation and inflammatory markers cannot be determined. Second, residual confounding may persist despite adjustment for multiple covariates, particularly from unmeasured inflammatory or metabolic factors. Third, participants were recruited from a single clinical site, which may limit the generalizability of the findings to other populations and settings. Fourth, HIV treatment duration and adherence may influence inflammatory markers; however, these factors were not fully captured in the present analysis. Fifth, the use of complete-case analysis may have introduced selection bias if missing TNF-α values were not missing completely at random. We did not formally compare the characteristics of participants included in the analytic sample (*n* = 227) with those excluded due to missing TNF-α (*n* = 139); therefore, we cannot exclude the possibility that selection bias influenced our findings. Multiple imputation was considered but not performed because the missingness mechanism could not be definitively established. These factors should be considered when interpreting the findings. Sixth, detailed information on antihypertensive medication classes (e.g., ACE inhibitors, angiotensin receptor blockers, mineralocorticoid receptor antagonists, and diuretics) was not systematically collected. In addition, the exclusion of participants with missing TNF-α measurements may have introduced selection bias if missing values were not completely random. While RAAS biomarkers were associated with TNF-α, these findings should be interpreted as indicative of relationships within a complex and interconnected inflammatory network rather than evidence of hierarchical biological dominance. History of tuberculosis was based on documented clinical records; however, detailed information on timing of TB diagnosis was not consistently available. Finally, restricting the analysis to individuals with complete data may further contribute to selection effects. These factors should be considered when interpreting the findings. Measurement of TNF-α at a single time point may also not fully capture temporal variations in inflammatory responses.

## Conclusion

In this cross-sectional study of Zambian adults with high HIV/TB burden, TNF-α levels were independently associated with RAAS activation (angiotensin II, aldosterone), HIV serostatus, dyslipidemia (VLDL, triglycerides), and specific inflammatory markers (sST2, IL-17 A, D-dimer). These results suggest that both residual HIV effects and neurohormonal/metabolic pathways contribute to inflammation in well-controlled HIV, with RAAS showing a relatively stronger association, though this should be interpreted cautiously. RAAS inhibitors, already widely used for hypertension, may represent a dual-purpose therapeutic strategy for mitigating inflammation-mediated cardiovascular risk in this setting. Prospective longitudinal studies and randomized controlled trials are needed to establish causality and test whether RAAS blockade reduces inflammation and improves clinical outcomes in this high-risk population.

## Supplementary Information

Below is the link to the electronic supplementary material.

Supplementary Material 1.

Supplementary Material 2.

Supplementary Material 3.

Supplementary Material 4.

Supplementary Material 5.

## Data Availability

The datasets used and/or analyzed during the current study are available from the corresponding author on reasonable request.

## Abbreviations

ART: Antiretroviral therapy
BMI: Body mass index
D-dimer: Fibrin degradation product D-dimer
ELISA: Enzyme-linked immunosorbent assay
HDL: High-density lipoprotein
HIV: Human Immunodeficiency Virus
hs-CRP: High-sensitivity C-reactive protein
IFN-γ: Interferon gamma
IL: Interleukin
IQR: Interquartile range
LDL: Low-density lipoprotein
LVMass: Left ventricular mass
MCV: Mean corpuscular volume
RAAS: Renin–angiotensin–aldosterone system
sST2: Soluble suppression of tumorigenicity 2
TB: Tuberculosis
Th17: T-helper 17 cells
TNF-α: Tumor necrosis factor alpha
VLDL: Very low-density lipoprotein
