# Supplementary material for: Clinical and neurohormonal correlates of plasma TNF-α in a Zambian outpatient cohort
Source: Discov Med (Singap). 2026 Nov 9;3(1):168. doi: 10.1007/s44337-026-00738-x (PMC13748043; doi:10.1007/s44337-026-00738-x)
Supplement: Supplementary file 1 [file 44337_2026_738_MOESM2_ESM.docx]

**Supplementary File 2**

**Structured Study Questionnaire**

**Sociodemographic, Clinical, and Behavioural Questionnaire — HAND Research Group Cohort Study**

**Participant ID:** ___________

**Date:** ***/***/_____

**Interviewer:** __________________

**Section A. Demographic Characteristics**

1. Age (years): ________
2. Sex

- ☐ Male
- ☐ Female

3. Marital status

- ☐ Married
- ☐ Single
- ☐ Divorced
- ☐ Widowed

4. Employment status

- ☐ Employed
- ☐ Self-employed
- ☐ Unemployed
- ☐ Student
- ☐ Retired

5. Highest level of education

- ☐ No formal education
- ☐ Primary
- ☐ Secondary
- ☐ College
- ☐ University

**Section B. Medical History**

6. Have you ever been diagnosed with hypertension?

- ☐ Yes
- ☐ No

If yes,

Year diagnosed: __________

7. Are you currently taking antihypertensive medication?

- ☐ Yes
- ☐ No

If yes, specify medication(s):

8. Have you ever been diagnosed with diabetes mellitus?

- ☐ Yes
- ☐ No

9. Have you ever been diagnosed with kidney disease?

- ☐ Yes
- ☐ No

10. Have you ever been diagnosed with cardiovascular disease?

- ☐ Yes
- ☐ No

Specify:

11. Have you ever been diagnosed with tuberculosis?

- ☐ Yes
- ☐ No

**Section C. HIV History**

12. HIV status

- ☐ Positive
- ☐ Negative

If HIV positive

13. Year diagnosed
14. Current ART regimen
15. Duration on ART

______ years

16. Have you missed any ART doses during the last month?

- ☐ Yes
- ☐ No

**Section D. Lifestyle Factors**

17. Do you currently smoke cigarettes?

- ☐ Yes
- ☐ No

**Section E. Medication Use**

18. Are you currently taking any of the following?

☐ ACE inhibitor

☐ ARB

☐ Diuretic

☐ Calcium channel blocker

☐ Beta blocker

☐ Statin

☐ Aspirin

☐ Other ____________________

**Section F. Clinical Measurements (Completed by Study Staff)**

| **Variable** | **Measurement** |
| --- | --- |
| Height (cm) | ______ |
| Weight (kg) | ______ |
| BMI (kg/m^2^) | ______ |
| Waist Circumference (cm) | ______ |
| Blood Pressure 1 | ______ |
| Blood Pressure 2 | ______ |
| Blood Pressure 3 | ______ |
| HIV status confirmed | Yes / No |
| ART regimen | ______ |
| Salt-sensitive (Yes/No) | ______ |
| Echocardiography completed | Yes / No |
| Blood sample collected | Yes / No |

**Interviewer Declaration**

I certify that the information recorded above is complete and accurate to the best of my knowledge.

Interviewer Name: ______________________

Signature: ______________________

Date: ____ / ____ / ______
