# Supplementary material for: Clinical and neurohormonal correlates of plasma TNF-α in a Zambian outpatient cohort
Source: Discov Med (Singap). 2026 Nov 9;3(1):168. doi: 10.1007/s44337-026-00738-x (PMC13748043; doi:10.1007/s44337-026-00738-x)
Supplement: Supplementary file 2 [file 44337_2026_738_MOESM3_ESM.docx]

**Supplementary file 3**

All variables were non-normally distributed (Shapiro–Wilk test, p < 0.05); therefore, continuous variables are presented as median (interquartile range [IQR]).

**Table 1. Normality Test Results for Continuous Variables**

| **Variable** | **Observations (n)** | **W** | **V** | **z** | **Prob>z** |
| --- | --- | --- | --- | --- | --- |
| Age (years) | 227 | 0.97204 | 4.659 | 3.563 | <0.001 |
| BMI (kg/m2) | 224 | 0.95597 | 7.252 | 4.585 | <0.001 |
| IL-6 (pg/mL) | 215 | 0.97735 | 3.599 | 2.957 | 0.002 |
| IL-17A (pg/mL) | 155 | 0.78832 | 25.333 | 7.34 | <0.001 |
| hs-CRP, (pg/mL) | 225 | 0.4005 | 99.123 | 10.638 | <0.001 |
| MCV (fL) | 153 | 0.77136 | 27.059 | 7.485 | <0.001 |
| IL-5 (pg/mL) IQR | 194 | 0.90158 | 14.299 | 6.111 | <0.001 |
| Soluble sT2, ng/mL | 153 | 0.75856 | 28.574 | 7.608 | <0.001 |
| Plasma Potassium (mmol/L) | 212 | 0.78005 | 34.524 | 8.173 | <0.001 |
| Spot Microalbumin, (mg/L) | 205 | 0.0504 | 144.747 | 11.461 | <0.001 |
| Aldosterone, (pg/mL) | 180 | 0.60582 | 53.665 | 9.115 | <0.001 |
| Renin, (pg/mL) | 211 | 0.7662 | 36.547 | 8.302 | <0.001 |
| Angiotensin, (pg/mL) | 155 | 0.56393 | 52.187 | 8.981 | <0.001 |
| VLDL(mmol/L) | 219 | 0.82847 | 27.698 | 7.677 | <0.001 |
| Cholesterol HDL Ratio | 194 | 0.84171 | 22.997 | 7.203 | <0.001 |
| LDL HDL Ratio | 194 | 0.97176 | 4.102 | 3.243 | 0.001 |
| Triglycerides (mmol/L) | 192 | 0.97252 | 3.956 | 3.158 | 0.001 |
| LVMass (g) | 195 | 0.84904 | 22.031 | 7.106 | <0.001 |
