# Supplementary material for: Clinical and neurohormonal correlates of plasma TNF-α in a Zambian outpatient cohort
Source: Discov Med (Singap). 2026 Nov 9;3(1):168. doi: 10.1007/s44337-026-00738-x (PMC13748043; doi:10.1007/s44337-026-00738-x)
Supplement: Supplementary file 3 [file 44337_2026_738_MOESM4_ESM.docx]

**Supplementary file 4**

**Table 1. Univariable linear regression analysis.**

| **Parameter** | **Beta (β)** | **β_std** | **95% L. Limit** | **95% U. Limit** | **p-value** | **SD Predictor** | **SD (TNF-a)** |
| --- | --- | --- | --- | --- | --- | --- | --- |
| Age, years | 53.6 | 0.11 | -8.2 | 115.5 | 0.089 | 13.84 | 6576.3 |
| Sex (Female) | 629.9 | 0.05 | -1052.4 | 5360.8 | 0.187 | 0.47 | 6576.3 |
| Body mass index (Kg/m^2^) | -128.1 | -0.11 | -281.6 | 25.3 | 0.101 | 5.67 | 6576.3 |
| Smoking status (Smoker) | -1647.2 | -0.05 | -5896.7 | 2602.3 | 0.446 | 0.21 | 6576.3 |
| History of TB (Yes) | 426.9 | 0.02 | -2315.7 | 3169.5 | 0.759 | 0.35 | 6576.3 |
| HIV status (Living with HIV) | 2573.3 | 0.19 | 803.9 | 4342.7 | **0.005** | 0.48 | 6576.3 |
| VLDL (mmol/L) | 4991.1 | 0.19 | 994.3 | 8987.9 | **0.015** | 0.24 | 6576.3 |
| Triglycerides (mmol/L) | 2238.6 | 0.19 | 486.7 | 3990.6 | **0.013** | 0.55 | 6576.3 |
| LDL HDL Ratio | 971.1 | 0.16 | 102.2 | 1840.1 | **0.029** | 1.10 | 6576.3 |
| Cholesterol HDL ratio | 974.0 | 0.18 | 164.0 | 1784.1 | **0.019** | 1.20 | 6576.3 |
| Angiotensin II (pg/mL) | 1.0 | 0.44 | 0.7 | 1.3 | **<0.001** | 2866.39 | 6576.3 |
| Aldosterone (pg/mL) | 0.8 | 0.42 | 0.6 | 1.1 | **<0.001** | 3484.31 | 6576.3 |
| Renin (pg/mL) | 0.7 | 0.34 | 0.3 | 1.0 | **<0.001** | 3217.04 | 6576.3 |
| hs-CRP (pg/mL) | 0.01 | 0.22 | 0.004 | 0.021 | **0.003** | 143678.80 | 6576.3 |
| D-dimer (pg/mL) | 0.04 | 0.24 | 0.02 | 0.06 | **<0.001** | 39432.83 | 6576.3 |
| IL-17A (pg/mL) | 1.3 | 0.24 | 0.6 | 2.0 | **<0.001** | 1204.55 | 6576.3 |
| IL-6 (pg/mL) | 43.2 | 0.38 | 23.9 | 62.7 | **<0.001** | 58.14 | 6576.3 |
| IL-5 (pg/mL) | 2.0 | 0.36 | 1.0 | 2.9 | **<0.001** | 1171.68 | 6576.3 |
| sST2 (pg/mL) | 108.8 | 0.33 | 68.5 | 149.0 | **<0.001** | 19.75 | 6576.3 |
| MCV (fL) | -101.0 | -0.19 | -180.2 | -21.7 | **0.013** | 12.09 | 6576.3 |
| Plasma Potassium (mmol/L) | 108.9 | 0.46 | 78.8 | 139.0 | **<0.001** | 28.01 | 6576.3 |
| Spot microalbumin (mg/L) | 13.7 | 0.13 | 0.5 | 26.9 | **0.042** | 62.22 | 6576.3 |
| LVMass (g) | -16.9 | -0.15 | -32.9 | -0.9 | **0.038** | 57.18 | 6576.3 |
| **Footnotes**: β = unstandardized coefficient (change in TNF-α in pg/mL per unit increase in predictor). β_std = standardized coefficient (change in SD of TNF-α per 1 SD increase in predictor). SD of TNF-α = 6576.3 pg/mL. Categorical variables were not standardized. **Abbreviations**: TNF-α, tumor necrosis factor-alpha; VLDL, very low-density lipoprotein; LDL, low-density lipoprotein; HDL, high-density lipoprotein; hs-CRP, high-sensitivity C-reactive protein; IL, interleukin; sST2, soluble suppression of tumorigenicity-2; MCV, mean corpuscular volume; LVMass, left ventricular mass. **Notes**: β coefficients represent the change in TNF-α concentration per unit increase in each predictor. Confidence intervals (CI) are reported at the 95% level. p-values were derived from unadjusted linear regression models. Continuous variables were analyzed per unit increase as indicated, and categorical variables are presented relative to the reference group. Statistical significance was defined as p < 0.05. | | | | | | | |

**Table 2. Multivariable linear regression analysis.**

| **Parameter** | **Beta (β)** | **β_std** | **95% L. Limit** | **95% U. Limit** | **p-value** | **SD Predictor** | **SD (TNF-a)** |
| --- | --- | --- | --- | --- | --- | --- | --- |
| Age, years | 19.3 | 0.04 | -58.7 | 97.4 | 0.626 | 13.84 | 6576.3 |
| Sex (Female) | 329.8 | 0.02 | -1678 | 2337.2 | 0.746 | 0.47 | 6576.3 |
| Body mass index (Kg/m^2^) | -158.2 | -0.14 | -332.9 | 16.4 | 0.075 | 5.67 | 6576.3 |
| Smoking status (Smoker) | -2183 | -0.07 | -6907 | 2542 | 0.363 | 0.21 | 6576.3 |
| History of TB (Yes) | -444 | -0.02 | -2958 | 2070.2 | 0.722 | 0.35 | 6576.3 |
| HIV status (Living with HIV) | 2581.1 | 0.19 | 439.4 | 4722.7 | **0.018** | 0.48 | 6576.3 |
| VLDL (mmol/L) | 4397.4 | 0.16 | 241.1 | 8553.7 | **0.038** | 0.24 | 6576.3 |
| Triglycerides (mmol/L) | 1971.6 | 0.17 | 152.7 | 3790.4 | **0.034** | 0.55 | 6576.3 |
| LDL HDL Ratio | 779.9 | 0.13 | -153.2 | 1713 | 0.101 | 1.10 | 6576.3 |
| Cholesterol HDL ratio | 803.1 | 0.15 | -71.38 | 1677.6 | 0.072 | 1.20 | 6576.3 |
| Angiotensin II (pg/mL) | 1.18 | 0.51 | 0.791 | 1.577 | **<0.001** | 2866.39 | 6576.3 |
| Aldosterone (pg/mL) | 0.884 | 0.47 | 0.588 | 1.18 | **<0.001** | 3484.31 | 6576.3 |
| Renin (pg/mL) | 0.276 | 0.14 | -0.166 | 0.717 | 0.219 | 3217.04 | 6576.3 |
| hs-CRP (pg/mL) | 0.0085 | 0.19 | 0.0008 | 0.0178 | 0.073 | 143678.80 | 6576.3 |
| D-dimer (pg/mL) | 0.037 | 0.22 | 0.014 | 0.059 | **0.001** | 39432.83 | 6576.3 |
| IL-17A (pg/mL) | 1.137 | 0.21 | 0.397 | 1.876 | **0.003** | 1204.55 | 6576.3 |
| IL-6 (pg/mL) | 12.98 | 0.11 | -28.64 | 54.61 | 0.538 | 58.14 | 6576.3 |
| IL-5 (pg/mL) | 0.671 | 0.12 | -0.716 | 2.058 | 0.34 | 1171.68 | 6576.3 |
| sST2 (pg/mL) | 104.4 | 0.31 | 48.16 | 160.7 | **<0.001** | 19.75 | 6576.3 |
| MCV (fL) | -77.62 | -0.14 | -179.2 | 23.98 | 0.133 | 12.09 | 6576.3 |
| Plasma Potassium (mmol/L) | 107.3 | 0.46 | 78.35 | 136.2 | **<0.001** | 28.01 | 6576.3 |
| Spot microalbumin (mg/L) | 12.24 | 0.12 | -4.943 | 29.42 | 0.161 | 62.22 | 6576.3 |
| LVMass (g) | -16.55 | -0.14 | -34.93 | 1.832 | 0.077 | 57.18 | 6576.3 |
| β = unstandardized coefficient (change in TNF-α in pg/mL per unit increase in predictor). β_std = standardized coefficient (change in SD of TNF-α per 1 SD increase in predictor). SD of TNF-α = 6576.3 pg/mL. Categorical variables (HIV status, TB history, sex, smoking) were not standardized; β_std for these variables represents the difference in SD units between groups. | | | | | | | |

**Table 3. Sensitivity analysis for Multivariable linear regression analysis.**

| **Parameter** | **Beta (β)** | **β_std** | **95% L. Limit** | **95% U. Limit** | **p-value** | **SD Predictor** | **SD (TNF-a)** |
| --- | --- | --- | --- | --- | --- | --- | --- |
| Age, years | 34.1 | 0.05 | -87.8 | 156.2 | 0.580 | 11.80 | 7984.9 |
| Sex (Female) | 843.9 | 0.00 | -1996.6 | 3684.5 | 0.558 | 0 | 7984.9 |
| Body mass index (Kg/m^2^) | -218.2 | -0.15 | -465.7 | 29.1 | 0.083 | 5.44 | 7984.9 |
| Smoking status (Smoker) | -2182.5 | -0.07 | -7795.5 | 3430.3 | 0.443 | 0.26 | 7984.9 |
| History of TB (Yes) | -444.2 | -0.02 | -3760.8 | 2872.4 | 0.791 | 0.40 | 7984.9 |
| VLDL (mmol/L) | 5620.7 | 0.18 | 13.3 | 11228.1 | **0.049** | 0.26 | 7984.9 |
| Triglycerides (mmol/L) | 2467.5 | 0.19 | 26.7 | 4908.3 | **0.048** | 0.60 | 7984.9 |
| LDL HDL Ratio | 1089.3 | 0.15 | -234.6 | 2413.4 | 0.106 | 1.10 | 7984.9 |
| Cholesterol HDL ratio | 1130.5 | 0.17 | -119.2 | 2380.2 | 0.076 | 1.19 | 7984.9 |
| Angiotensin II (pg/mL) | 1.20 | 0.40 | 0.73 | 1.67 | **<0.001** | 2683.64 | 7984.9 |
| Aldosterone (pg/mL) | 0.88 | 0.39 | 0.53 | 1.23 | **<0.001** | 3554.77 | 7984.9 |
| Renin (pg/mL) | 0.27 | 0.12 | -0.28 | 0.83 | 0.331 | 3517.58 | 7984.9 |
| hs-CRP (pg/mL) | 0.01 | 0.18 | -0.001 | 0.018 | 0.077 | 141127.10 | 7984.9 |
| D-dimer (pg/mL) | 0.03 | 0.18 | 0.01 | 0.06 | **0.007** | 47655.77 | 7984.9 |
| IL-17A (pg/mL) | 1.13 | 0.21 | 0.25 | 2.01 | **0.011** | 1449.17 | 7984.9 |
| IL-6 (pg/mL) | 14.9 | 0.07 | -40.7 | 70.5 | 0.595 | 37.58 | 7984.9 |
| IL-5 (pg/mL) | 0.67 | 0.10 | -1.09 | 2.43 | 0.451 | 1163.26 | 7984.9 |
| sST2 (pg/mL) | 104.9 | 0.27 | 38.1 | 171.7 | **0.002** | 20.28 | 7984.9 |
| MCV (fL) | -103.3 | -0.15 | -242.4 | 35.7 | 0.143 | 11.86 | 7984.9 |
| Plasma Potassium (mmol/L) | 107.2 | 0.48 | 72.5 | 142.1 | **<0.001** | 35.58 | 7984.9 |
| Spot microalbumin (mg/L) | 12.7 | 0.11 | -9.0 | 34.5 | 0.247 | 71.55 | 7984.9 |
| LVMass (g) | -27.4 | -0.17 | -55.5 | 0.62 | 0.055 | 50.59 | 7984.9 |
| **Footnote**: β = unstandardized coefficient (change in TNF-α in pg/mL per unit increase in predictor). β_std = standardized coefficient (change in SD of TNF-α per 1 SD increase in predictor). This model includes an expanded set of covariates to assess robustness of associations. Variables were selected based on biological relevance; however, given the number of covariates relative to sample size, results should be interpreted cautiously. | | | | | | | |
