# Supplementary material for: Clinical and neurohormonal correlates of plasma TNF-α in a Zambian outpatient cohort
Source: Discov Med (Singap). 2026 Nov 9;3(1):168. doi: 10.1007/s44337-026-00738-x (PMC13748043; doi:10.1007/s44337-026-00738-x)
Supplement: Supplementary file 4 [file 44337_2026_738_MOESM5_ESM.docx]

**Supplementary file 5**

In sensitivity analysis using natural log-transformed TNF-α and log-transformed significant predictors. Both TNF-α and all statistically significant continuous predictors were natural log-transformed; HIV serostatus was retained as a binary variable. β coefficients represent changes in ln(TNF-α) per unit increase in ln(predictor) and are dimensionless. Most associations (VLDL, triglycerides, aldosterone, D-dimer, IL-17A, sST2) remained consistent in direction and significance, supporting robustness to skewness. The angiotensin II coefficient attenuated to zero owing to the wide dynamic range of values on the log scale (a scaling artifact, not a loss of association). The HIV serostatus association reversed direction and the plasma potassium association attenuated to borderline non-significance, indicating sensitivity to model specification; these findings are acknowledged as limitations. Models were adjusted for age, sex, BMI, smoking status, HIV serostatus, and history of TB.

**Table 1. Sensitivity analysis using natural log-transformed TNF-α and log-transformed significant predictors.**

| **Parameter** | **Beta (β)** | **95% L. Limit** | **95% U. Limit** | **p-value** | **Consistency with Primary Analysis** |
| --- | --- | --- | --- | --- | --- |
| HIV status (Living with HIV) | -0.31 | -0.62 | -0.01 | **0.042** | Direction reversed (see note below) |
| VLDL (mmol/L) | 0.74 | 0.08 | 1.41 | **0.029** | **Consistent (positive, significant)** |
| Triglycerides (mmol/L) | 0.38 | 0.09 | 0.67 | **0.010** | **Consistent (positive, significant)** |
| Angiotensin II (pg/mL) | 0.00 | 0.00 | 0.00 | **<0.001** | Attenuated to zero (see note below) |
| Aldosterone (pg/mL) | 1.11 | 0.91 | 1.23 | **<0.001** | **Consistent (positive, significant)** |
| D-dimer (pg/mL) | 0.64 | 0.33 | 0.96 | **<0.001** | **Consistent (positive, significant)** |
| IL-17A (pg/mL) | 0.96 | 0.78 | 1.13 | **<0.001** | **Consistent (positive, significant)** |
| sST2 (pg/mL) | 0.04 | 0.03 | 0.05 | **<0.001** | **Consistent (positive, significant)** |
| Plasma Potassium (mmol/L) | 0.01 | -0.00 | 0.01 | 0.053 | Attenuated to borderline non-significance (see note below) |
| **Abbreviations**: TNF-α, tumor necrosis factor-alpha; VLDL, very low-density lipoprotein; IL, interleukin; sST2, soluble suppression of tumorigenicity-2**.** | | | | | |
